# Preparation of Functional Monomers as Precursors of Bioprobes from a Common Styrene Derivative and Polymer Synthesis

**DOI:** 10.3390/molecules23112875

**Published:** 2018-11-04

**Authors:** Riho Hayama, Tetsuo Koyama, Takahiko Matsushita, Ken Hatano, Koji Matsuoka

**Affiliations:** 1 Division of Material Science, Graduate School of Science and Engineering, Saitama University, Sakura, Saitama 338-8570, Japan; ri11270825@gmail.com (R.H.); koyama@fms.saitama-u.ac.jp (T.K.); takahiko@fms.saitama-u.ac.jp (T.M.); khatano@fms.saitama-u.ac.jp (K.H.); 2 Medical Innovation Research Unit (MiU), Advanced Institute of Innovative Technology (AIIT), Saitama University, Sakura, Saitama 338-8570, Japan

**Keywords:** styrene, glycopolymers, radical polymerization, lectins, fluorescence spectrometry

## Abstract

CM-Str (4-(Chloromethyl)styrene) was used as a useful starting material for the construction of a series of functional monomers. Substitution of the chlorine to the corresponding azide was performed, and the reduction of the azide proceeded smoothly to afford an aminostyrene, which was used as a common precursor for the preparation of functional monomers. Condensation of the amine with a fluorophore, biotin and carbohydrate was accomplished. Among the monomers, a carbohydrate monomer was polymerized with or without acrylamide as a model polymerization to yield the corresponding water-soluble glycopolymers, and biological evaluations of the glycopolymers for a lectin, and wheat germ agglutinin (WGA), were carried out on the basis of the fluorescence change of tryptophan in the WGA.

## 1. Introduction

Although oligosaccharides and their derivatives are of great interest as bioactive compounds in various research areas, the affinities of the carbohydrate moieties are not so high [1,2]. In nature, the oligosaccharide chains in glycoconjugates play a role and have high specificities in biological events, and a recent report suggested that the affinities of oligosaccharide chains in glycolipids and glycoproteins were enhanced by the self-assembly of glycoconjugates, which display multivalent-type clusters. Much attention has been paid to the formation of synthetic glycoclusters by covalent bonds, and glycodendrimers [3,4,5] and glycopolymers [6,7,8,9,10] have been successfully developed. In our ongoing studies of glycopolymers, we have reported a conventional method for the preparation of a glycopolymer by means of Huisgen cycloaddition as a key reaction for the synthesis of monomers and glycopolymers [11]. The efficient assembly of carbohydrate moieties induced an enhancement of the affinities for a lectin on the basis of carbohydrate-protein interactions [12,13,14,15].

The functionalization of monomers as bioprobes is highly attractive and of great importance in order to evaluate their biological phenomena. For example, fluorogenic monomers are useful in order to evaluate an interaction between carbohydrate molecules and a glyscosidase [16]. In addition to the fluorogenic compounds, the biotin-avidin interaction is also an important biological event and a useful biological tool for biochemical and biomedical uses [17,18]. In this paper, we describe an alternative pathway for the convenient preparation of a glycomonomer and functional monomers using a commercially available and inexpensive reagent, 4-chloromethylstyrene (CM-Str), as a starting material. The further transformation of the glycomonomer into corresponding glycopolymers is reported. In addition to the syntheses, the results of the biological evaluations of the glycopolymers with *N*-acetyl-d-glucosamine (NAG) residues for wheat germ agglutinin (WGA) [19] as a model lectin are also described.

## 2. Results and Discussion

The utilization of 4-(chloromethyl)styrene **1** (CM-Str) as a starting material for the preparation of a series of functionalized monomers was examined in order to construct polymeric bioprobe families. We previously reported a convenient synthesis of glycomonomers using CM-Str **1** as a starting material by means of the Huisgen cycloaddition reaction [20,21,22,23] and polymer syntheses [11]. In this paper, alternative preparations of a glycomonomer and other useful functional monomers are described.

### 2.1. Conversion of 4-(Chloromethyl)styrene into an Amine as a Common Precursor

**Scheme 1:**
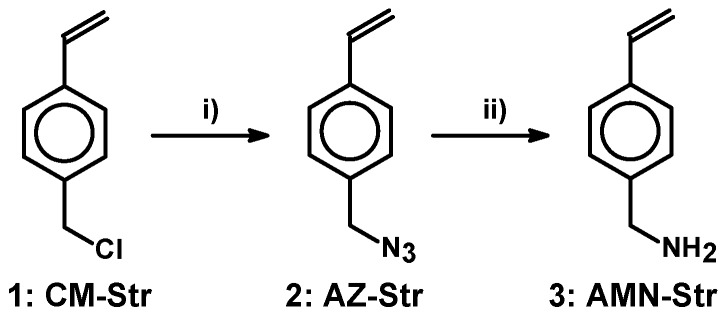
Reagents and conditions: (i) NaN_3_, DMF, 80 °C, 1 h, (ii) TPP, H_2_O, THF, 0 °C, 2.5 h.

Commercially available CM-Str **1** as a versatile precursor for an azide compound was used as a convenient starting material, and the conversion of the azide **2** (AZ-Str) [24] into the corresponding amine derivative by the Staudinger reaction was performed. Scheme 1 summarizes the preparation of aminostyrene derivative **3** (AMN-Str) [25] from CM-Str **1**. The S_N_2 replacement of the chloride of CM-Str **1** was performed by an azide anion in a polar aprotic solvent at a slightly high temperature to afford the corresponding AZ-Str **2** in 90% yield [11]. The Staudinger reaction of AZ-Str **2** with triphenylphosphine (TPP) afforded the corresponding CM-Str **3** in 96% yield. Two-step-conversion from **1** to **3** was reported and the yield was moderate (63% in two steps) [25]. In our case, the yield was improved (86% in two steps) by the replacement of a solvent from benzene to THF in Scheme 1(ii).

### 2.2. Synthetic Conversion of the Amine into Functionalized Monomers

**Scheme 2:**
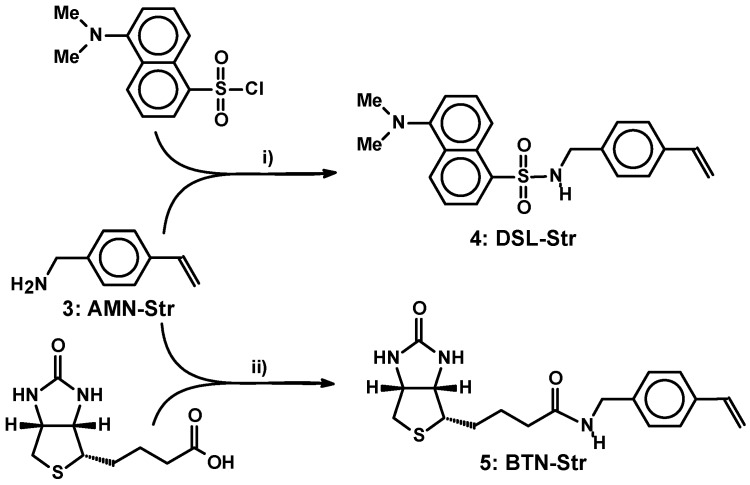
Reagents and conditions*:* (i) Dansyl chloride, Et_3_N, dichloromethane (DCM), rt, 5.5 h, (ii) biotin, DMT-MM, DMF, rt, 1.5 h.

Given the success of the simple preparation of AMN-Str **3**, which had a highly reactive primary amino function, we next turned our attention to the applicability of AMN-Str **3** as a common precursor for the synthesis of functional monomers. Scheme 2 shows the chemical conversion of AMN-Str **3** into useful monomers with a styrene moiety. Commercially available acid chloride of a dansyl derivative was first used to introduce fluorescent moieties into the synthetic polymer. Thus, the dansylation of AMN-Str **3** with dansyl chloride in the presence of an acid scavenger proceeded smoothly to afford the dansylated styrene derivative **4** (DSL-Str) [26] as a fluorogenic monomer in 76% yield. Structural elucidations were performed by a combination of IR, ^1^H-NMR and elemental analyses. An alternative functionalization of AMN-Str **3** was performed using a biotin as an attractive biomolecule in order to obtain a strong interaction between an avidin and the biotin [18]. Thus, the d-(+)-biotin was coupled with AMN-Str **3** using typical condensing reagents [27] such as 1-ethyl-3-(3-dimethylaminopropyl)carbodiimide hydrochloride (EDC), *N*,*N*′-diisopropyl carbodiimide (DIC), and (benzotriazol-1-yloxy)tris(dimethylamino)phosphonium hexafluorophosphate (BOP). All of the coupling reagents unfortunately produced unsuccessful or poor results due to a solubility problem of the biotin derivatives. The results are summarized in Table 1. Since well-known condensing reagents did not work in this coupling reaction, we decided to use 4-(4,6-dimethoxy-1,3,5-triazin-2-yl)-4-methylmorpholinium chloride (DMT-MM), which was introduced by Kunishima et al. [28]. The biotin was treated with AMN-Str **3** in the presence of DMT-MM to afford the corresponding amide **5** (BTN-Str) in 91% yield.

### 2.3. Preparation of a Carbohydrate Monomer

**Scheme 3:**
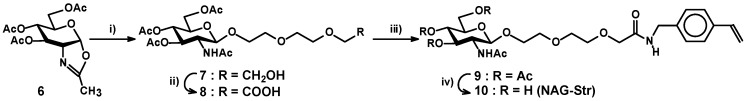
Reagents and conditions: (i) 2,2′-[ethane-1,2-diylbis(oxy)]di(ethan-1-ol), CSA, ClCH_2_CH_2_Cl, 90 °C, 1 h, (ii) aq. NaHCO_3_, KBr, TEMPO, trichloroisocyanuric acid, acetone, 0 °C, 20 min, then rt, 4.5 h; (iii) AMN-Str **3**, DMT-MM, DCM, rt, 3.5 h; (iv) NaOMe, MeOH, rt, 1.5 h.

Although the effective conversion of the aminostyrene **3** (AMN-Str) into functional monomers was accomplished, the further transformation of the amine was required in order to produce carbohydrate monomers, which are bioactive compounds and are recognized by various lectin molecules. A schematic diagram of the preparation of a glycomonomer **10** (NAG-Str) is shown in Scheme 3. Since the hydrophilicity of the styrene moiety was low, we decided to use a polyethyleneoxy alcohol, which has the appropriate hydrophilicities as an aglycon moiety. Thus, a known oxazoline derivative **6** [29] was treated with 2,2′-(ethylenedioxy)diethanol in the presence of camphorsulfonic acid (CSA) [30] to yield a monoalcoholic glycoside **7** as a yellow syrup in 76% yield. An anomeric configuration of glycoside **7** was determined using ^1^H-NMR spectra. The results revealed a signal assignable to H-1 at δ 4.77 as a doublet with *J*_1,2_ = 8.60 Hz, which was confirmed to be a β-glycoside. The primary alcohol had to be oxidized to carboxylic acid before the coupling reaction of the glycoside with the amine **3**. Thus, 2,2,6,6-tetramethylpiperidine 1-oxyl free radical (*TEMPO*)-mediated oxidation [31] of the alcohol **7** was carried out to produce the desired carboxylic acid **8**, which was directly used for the next step due to the production of an inseparable byproduct in the reaction. We therefore estimated the yield of the oxidation to be approximately 46%. Freshly prepared carboxylic acid **8** and the amine **3** were applied for a DMT-MM-mediated coupling reaction, and the reaction proceeded smoothly to produce the corresponding styrene derivative **9** as a colorless syrup in 78% yield after chromatographic purification. Structural elucidation of product **9** was performed by means of ^1^H- and ^13^C-NMR spectroscopic analyses. Since the appearance of aromatic ring proton signals at 7.39 ppm and 7.27 ppm in **9** was observed in the ^1^H-NMR results, the progress of the coupling reaction was confirmed. In addition to the ^1^H-NMR results, the combined results of the ^13^C-NMR and HMQC experiments also supported the formation of **9**. The removal of the acetyl protection in **9** was performed by using the Zemplén method [32] to afford NAG-Str **10** as a water-soluble material in 99% yield.

### 2.4. Polymerization of a Glycomonomer by Means of Radical Polymerization

**Scheme 4:**
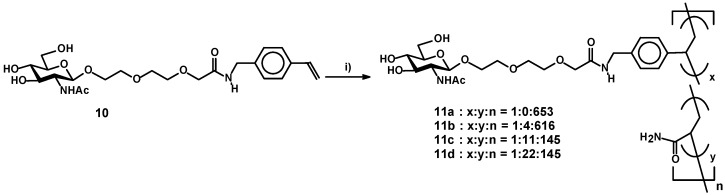
Reagents and conditions: (i) acrylamide, ammonium persulfate (APS), tetramethylethylenediamine (TEMED) rt.

The synthetic conversion of AMN-Str **3** into functional monomers was successfully demonstrated, and our next objective was the chemical conversion of the monomers into the corresponding polymers. In our synthetic study of glycoclusters, radical polymerization was applied for the synthetic assembly of carbohydrate epitopes. This type of monomer had not hitherto been used in our study. Thus, the *N*-acetyl-d-glucosaminyl monomer **10** (NAG-Str) was used as a model monomer for the preparation of glycopolymers, as shown in Scheme 4. The polymerization ability of NAG-Str **10** was first evaluated by radical polymerization without a comonomer in aqueous media at room temperature. NAG-Str **10** underwent homopolymerization to yield white powdery **11a** in 71% yield after dialysis against water followed by lyophilization. A successful polymerization protocol was applied for the preparation of copolymers of NAG-Str **10** and acrylamide, and the results of the copolymerizations are summarized in Table 2. The weight-average molecular weights ($\bar{Mw}$) of the glycopolymers were estimated by size exclusion chromatography in 0.3 M aqueous NaCl solution. From the weight-average molecular weight ($\bar{Mw}$) of glycopolymer **11a**, the degree of polymerization was estimated to be 653. The polymer compositions of glycopolymers **11b**, **11c** and **11d** were estimated on the basis of the ^1^H-NMR results. All of the polymers (**11a**–**11d**) had water-soluble abilities and appropriate molecular weights as estimated by size-exclusion chromatography (SEC) analyses.

### 2.5. Biological Evaluations of the Glycopolymers for Lectin

**Figure 1:**
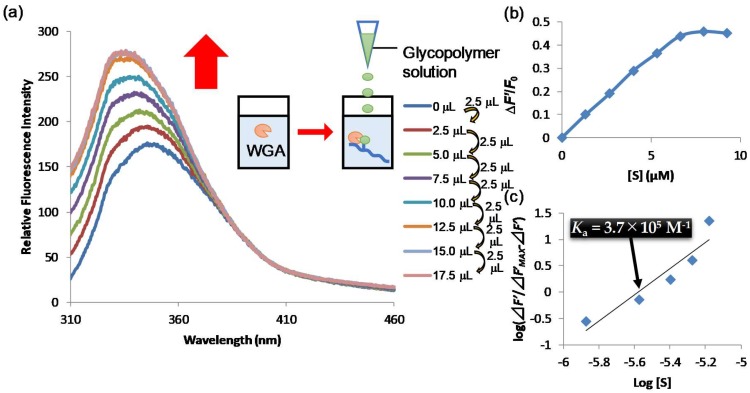
Biological evaluations of the WGA–carbohydrate interaction of **11b**. (**a**) Changes in fluorescence emission spectra of WGA (0.65 μM, 3.0 mL, 50 mM Tris-HCl buffer containing 150 mM NaCl, pH 7.4, 4 °C) upon addition of 2.5-μL aliquots of glycopolymer **11b** (74.5 μM). (**b**) Plots of Δ*F’*/*F*_0_ versus [S], where Δ*F’* is the change in the intensity at 348 nm of WGA with various concentrations of **11b**, *F*_0_ is the intensity of WGA alone, and [S] is total ligand concentration. (**c**) Hill plots of log [Δ*F*′*/(*Δ*F*′*_MAX_* − Δ*F*′)] versus log [S].

Once the synthetic assembly of sugar moieties using polymer chemistry was accomplished, we turned our attention to the biological properties of the glycopolymers. Interactions between glycopolymers **11a**–**11d** and WGA, which showed high binding specificity for *N*-acetyl-d-glucosamine (NAG) and its oligomers [33,34], were therefore evaluated on the basis of fluorescence measurements. Fluorescence measurements of WGA and polymers with NAG residues were preliminarily performed in 0.65 μM of WGA solution in 50 mM Tris-HCl buffer (pH 7.4) at 4 °C [11]. The results of the measurement using **11b** and the analyses of the results are shown in Figure 1 as an example. The intensity of the peaks at 348 nm gradually increased upon addition of NAG polymer solutions, as shown in Figure 1a. The difference between the wavelengths on the maximum relative fluorescence intensity of WGA alone and WGA with the saturated concentration of a glycopolymer is represented as Δλ. From the results in Figure 1a, we can see that Δλ was −14.2 nm, as shown in Table 3. These phenomena occurred due to the change in the environment around the tryptophan residue located at or near the binding sites of WGA. The changes in fluorescence intensity were substituted into the Hill equation [35]:

(1) $$\log\left(\frac{\Delta F}{\Delta F_{MAX}-\Delta F}\right)=\log\left[S\right]+\log K_{a}$$

where Δ*F* (Δ*F* = *F* − *F*_0_) is the difference in relative fluorescence intensity at 348 nm of WGA between *F*, which is the fluorescence intensity of WGA with various concentrations of a glycopolymer and *F*_0_, which is fluorescence intensity of a solution of WGA itself (without a glycopolymer); and Δ*F_MAX_* is the difference between *F_MAX_*, which is the final fluorescence intensity at the saturated concentration of a glycopolymer, and *F*_0_.

The influence of the dilution of the WGA concentration by the addition of a glycopolymer solution was corrected using the following equation:

(2) $$\Delta F\prime =\frac{V}{V_{0}}\Delta F$$

where *V*_0_ is the initial volume of the WGA solution and *V* is the measured volume of the WGA solution with a given glycopolymer concentration based on the sugar unit in the glycopolymer [S]; Equation (1), therefore, is represented as

(3) $$log\left(\frac{\Delta F\prime }{\Delta F\prime_{MAX}-\Delta F\prime }\right)=log\left[S\right]+logK_{a}$$

When Δ*F*’/*F*_0_ was plotted versus [*S*], as shown in Figure 1b, an enhancement of the relative fluorescence intensity by about 46% was observed, as shown in Table 3. From the results of a Hill plot of *log*[Δ*F*/(Δ*F_MAX_* − Δ*F*)] versus *log*[*S*], as shown in Figure 1c, the association constant K_a_ was estimated to be 3.7 × 10^5^ M^−1^. The results for other glycopolymers against WGA are summarized in Table 3. Association constants were estimated to be 1.8–4.2 × 10^5^ M^−1^. The association constant *K_a_* was the largest when copolymer **11d** was used. This can be explained by the suitable density of the carbohydrate moieties on the glycopolymer, as the ligand was possibly the best fit for the location of each binding site on WGA [36]. However, their *K_a_* values were nearly in the same molar range. In addition to the fluorometric assays for the glycopolymers, biological evaluations of some of the glycopolymers and WGA were carried out by means of surface plasmon resonance (SPR) [37]. The results of the SPR analyses showed that the *K_a_* for **11b** was 3.30 × 10^5^ M^−1^; for **11c** it was 3.41 × 10^5^ M^−1^. These *K_a_* values were in good agreement with the results from the fluorometric assay measurements in this study. From the results of the biological evaluation of the glycopolymers for lectin, we can see that these types of glycopolymers also have a sugar-clustering effect [38,39,40].

## 3. Experimental

### 3.1. Materials and Methods

Unless otherwise stated, all commercially available solvents and reagents were used without further purification. *N*,*N*-Dimethylformamide (DMF) was stored over molecular sieves (4 Å MS) and methanol (MeOH) was stored over 3 Å MS before use. Acrylamide was recrystallized from benzene before use. Optical rotations were determined with a JASCO DIP-1000 digital polarimeter (JASCO Corp., Tokyo, Japan). IR spectra were obtained using a Shimadzu IR Prestige-21 spectrometer (Shimadzu Corp., Kyoto, Japan). ^1^H-NMR spectra were recorded at 400 MHz for ^1^H and at 100 MHz for ^13^C with a Bruker DPX-400 spectrometer or at 500 MHz for ^1^H and at 125 MHz for ^13^C with a Bruker AVANCE 500 spectrometer (Bruker BioSpin, Ettlingen, Germany) in chloroform-*d* (CDCl_3_) or deuterium oxide (D_2_O). Chemical shifts are expressed as parts per million (ppm, δ) and are relative to an internal tetramethylsilane (TMS) in CDCl_3_ (δ 0.0) or HDO in D_2_O (δ 4.78) for ^1^H and CHCl_3_ in CDCl_3_ (δ 77.0), CH_3_ in MeOD (δ 49.0) or CH_3_ in acetone (δ 215.0) for ^13^C. Ring-proton assignments in the ^1^H-NMR spectra were made by first-order analysis of the spectra and are supported by the results of homonuclear decoupling experiments and H-H or HMQC experiments. Elemental analyses were performed with a Fisons EA1108 (Thermo Fisher Scientific Inc., Waltham, MA, USA) on samples that were extensively dried at 50–60 °C over phosphorus pentoxide for 4–5 h. Matrix-assisted laser desorption/ionization time-of-flight mass spectra (MALDI-TOF-MS) were obtained using a Bruker AutoflexIII spectrometer (Bruker Daltonics, Bremen, Germany). Reactions were monitored by thin layer chromatography (TLC) on a precoated plate of silica gel 60F_254_ (layer thickness, 0.25 mm; E. Merck, Darmstadt, Germany). For the detection of the intermediates, TLC sheets were (a) dipped in a solution of 85:10:5 (*v*/*v*/*v*) MeOH-*p*-anisaldehyde-concd H_2_SO_4_ and heated for a few minutes (for carbohydrate); or (b) dipped in an aq solution of 5wt% KMnO_4_ and heated similarly (for detection of C=C double bonds). Column chromatography was performed on silica gel (Silica Gel 60; 63–200 μm, E. Merck, Darmstadt, Germany). Flush column chromatography was also performed on silica gel (Silica Gel 60, spherical neutral; 40–100 μm, E. Merck, Darmstadt, Germany). All extractions were concentrated below 45 °C under diminished pressure. Dialysis was performed against distilled water using a dialysis tubing [molecular cutoff (MWCO): 12 k–16 kDa, Dow Chemical Co., Midland, MI, USA]. Wheat germ agglutinin (WGA; a lectin from *Triticum vulgaris*) was purchased from J-Oil Mills (Lot # 62015) (J-Oil Mills, Inc., Tokyo, Japan).

### 3.2. Synthesis

#### 3.2.1. 4-Azidomethylstyrene (AZ-Str) (**2**)

To a solution of chloromethyl styrene **1** (CM-Str) (10.0 g, 65.5 mmol) in DMF (50 mL) was added sodium azide (12.4 g, 190 mmol) and the mixture was stirred at 80 °C for 1.0 h. After the complete vanishing of **1** judged by TLC, the reaction mixture was evaporated in vacuo. The residue was diluted with CHCl_3_ and was then washed successively with water and brine and dried over anhyd MgSO_4_. The organic solution was filtered and evaporated in vacuo. The residual syrup was applied to a column of silica gel with 40:1 (*v*/*v*) hexane—EtOAc as the eluent to give pure AZ-Str **2** (9.40 g, 90.1%) as a yellow syrup: R_f_ 0.28 [hexane]; IR (NEAT) 3088 (νC–H, Ph), 2930 (ν_C-H_), 2097 (ν_N=N=N_), 1630 (ν_C=C_) cm^−1^; ^1^H-NMR (500 MHz, CDCl_3_): δ 7.43 (d, 2 H, *J* = 8.15 Hz, Ph), 7.28 (d, 2 H, *J* = 8.15 Hz, Ph), 6.72 (dd, 1 H, *J*_cis_ = 10.85 Hz, *J*_trans_ = 17.60 Hz, CH=), 5.77 [dd, 1 H, *J*_gem_ = 0.50 Hz, trans of CH_2_=], 5.28 [dd, 1 H, cis of CH_2_=], 4.32 (s, 2 H, PhCH_2_).

#### 3.2.2. Aminomethylstyrene (AMN-Str) (**3**) 

To a solution of AZ-Str **2** (3.00 g, 18.8 mmol) in anhydr THF (10 mL) was added triphenyl phosphine (TPP) (4.94 g, 20.7 mmol). H_2_O (30 mL) was added to the solution at 0 °C and the mixture was stirred at room temperature for 2.5 h. After the complete transformation of the AZ-Str **2** judged by TLC, the reaction mixture was diluted with CHCl_3_ and the mixture was shaken with 3 M aq H_2_SO_4_. The extraction was partitioned and the aqueous layer was treated with NaOH until ca. pH 12 to produce precipitates. To the mixture was added CHCl_3_ and the organic solution was washed with brine, dried over anhyd MgSO_4_, and evaporated to give pure AMN-Str **3** [24] (2.40 g, 95.6%) as a yellow syrup: *R*_f_ 0.37 [5:5:1 (*v*/*v*/*v*) CHCl_3_—MeOH—water]; IR (NEAT) 3281 (ν_N‒H_), 3005 (ν_C-H_, Ph), 1628 (δ_N‒H_) cm^−1^; ^1^H-NMR (500 MHz, CDCl_3_): δ 7.39 (d, 2 H, *J* = 8.10 Hz, Ph), 7.27 (d, 2 H, *J* = 9.15 Hz, Ph), 6.71 (dd, 1 H, *J_cis_* = 10.90 Hz, *J_trans_* = 17.60 Hz, CH=), 5.73 (dd, 1 H, *J*_gem_ = 0.83 Hz, *trans* of CH_2_=) 5.28 (dd, 1 H, *cis* of CH_2_=), 3.86 (s, 2 H, PhCH_2_), 1.56 (s, 2 H, NH_2_).

#### 3.2.3. 4-{[5-(Dimethylamino)-1-naphthalenesulfonamido]methyl}styrene (DSL-Str) (**4**) 

To a stirred solution of AMN-Str **3** (512 mg, 3.8 mmol) and Et_3_N (0.53 mL, 456 mmol) in dichloromethane (50 mL) a solution of dansyl chloride (1.23 g, 4.56 mmol) in dichloromethane (40 mL) was added dropwise at room temperature under a N_2_ atmosphere. After stirring at room temperature for 5.5 h, the reaction mixture was evaporated in vacuo. The resulting mixture was diluted with CHCl_3_ and was then washed successively with water and brine and dried over anhyd MgSO_4_. The organic mixture was filtered and evaporated in vacuo. The residual syrup was applied to a column of silica gel with 5:2 (*v*/*v*) hexane—EtOAc as the eluent to give pure DSL-Str **4** [26] (1.052 g, 75.5%) as yellow crystals: mp 103.0‒103.4 °C; *R*_f_ 0.40 [5:2 (*v*/*v*) hexane—EtOAc]; IR (NEAT): 3286.70, 3273.20 (ν_N-H_), 3084.18 (ν_C-H_, Ph), 1625.99 (δ_N-H_), 1305.81 (ν_C-N_), 1066.64 (ν_S=O_) cm^−1^; ^1^H-NMR (500 MHz, CDCl_3_): δ 8.53 (d, 2 H, *J* = 8.50 Hz, Naph), 8.27 (t, 2 H, *J* = 8.85 Hz, *J* = 7.45 Hz, Naph), 7.53 (dt, 2 H, *J* = 10.90 Hz, *J* = 17.60 Hz, Naph), 7.19 (d, 2 H, *J* = 7.95 Hz, Ph) 7.19 (d, 1 H, *J* = 6.35 Hz, Naph), 7.01 (d, 2 H, *J* = 7.80 Hz, Ph), 6.60 (dd, 1 H, *J* =10.87 Hz, *J* = 17.53 Hz, Naph), 5.66 (br dd, 1 H, *J* = 17.61 Hz, Naph), 5.20 (br dd, 1 H, *J* = 10.85 Hz, Naph), 4.86 (t, 2 H, *J* = ~6 Hz, NH), 4.06 (d, 2 H, *J* = 6.05 Hz, CH_2_), 2.89 (s, 6 H, NCH_3_ × 2) (^1^H-MNR spectrum can be found in Supplementary Materials).

#### 3.2.4. 4-({5-[(3aR,6S,6aS)-2-Oxo-1,3,3a,4,6,6a-hexahydrothieno[3,4-d]imidazol-6-yl]pentanamido}methyl)-styrene (BTN-Str) (**5**)

To a stirred solution of AMN-Str **3** (60.0 mg, 0.45 mmol) and biotin (121 mg, 495 mmol) in DMF (1 mL) was added DMT-MM (150 mg, 0.54 mmol) at room temperature under a N_2_ atmosphere. After stirring at room temperature for 1.5 h, the white suspension was poured into ice-cold water and extracted with EtOAc. When extraction with EtOAc was performed, an insoluble mass was also observed. The organic solution and the insoluble mass were successively washed with ice-cold water, satd aq NaHCO_3_ and brine, and the whole mixture was filtrated to give a white amorphous powder BTN-Str **5** (148 mg, 91.3%): *R*_f_ = 0.53 [5:1 (*v*/*v*) CHCl_3_—MeOH]; ^1^H-NMR (500 MHz, DMSO-*d*_6_): δ 8.34 (br s, 1 H, NHCH_2_), 7.41 (d, 2 H, *J* = 8.15 Hz, Ph), 7.21 (d, 2 H, *J* = 8.15 Hz, Ph), 6.71 (dd, 1 H, *J_cis_* = 10.95 Hz, *J_trans_* = 17.65 Hz, CH=CH_2_), 6.42 [br s, 1 H, NH (biotin)], 6.37 [s, 1 H, NH (biotin)], 5.79 (dd, 1 H, *J*_gem_ = 0.70 Hz, *trans* of CH2=), 5.22 (d, 1 H, *cis* of CH_2_=), 4.30 [dd, 1 H, *J* =5.15 Hz, *J* =7.65 Hz, CHCH_2_ (biotin)], 4.24 (d, 2 H, *J* = 5.95 Hz, PhCH_2_), 4.12 (ddd, 1 H, *J* = 1.80 Hz, *J* = 4.35 Hz, *J* = 7.45 Hz, NHC*H*CH), 3.09 (ddd, 1 H, *J* = 2.55 Hz, *J* = 4.35 Hz, *J* = 10.5 Hz, SCH), 2.82 (dd, 1 H, *J*_gem_ = 12.4 Hz, SCH_a_), 2.58 (d, 1 H, SCH_b_), 2.14 (t, 2 H, *J* =7.45 Hz, CH_2_CO), 1.5 (m, 4 H, CH_2_ × 2) (^1^H-MNR spectrum can be found in Supplementary Materials).

#### 3.2.5. 2-[2-(2-Hydroxyethoxy)-ethoxy]-ethyl-*O*-2-acetamido-3,4,6-tri-*O*-acetyl-2-deoxy-β-d-glucopyranoside (**7**)

The quantitative preparation of an oxazoline **6** was accomplished from 2-acetamido-1,3,4,6-tetra-*O*-acetyl-α-d-glucosamine (2.00 g, 5.14 mmol) by the method previously reported [29]. To a solution of oxazoline and 2,2′-[ethane-1,2-diylbis(oxy)]di(ethan-1-ol) (3.4 mL, 25.6 mmol) in dichloroethane (17 mL) was added CSA (0.12 g, 0.51 mmol) under a N_2_ atmosphere and the reaction mixture was stirred at 90 °C for 1 h. After cooling the reaction mixture, CHCl_3_ was added. The organic mixture was successively washed with water, satd aq NaHCO_3_ and brine, dried over anhyd MgSO_4_, filtered, and evaporated to yield a dark yellow syrup, which was applied to a column of silica gel with 20:1 (*v*/*v*) CHCl_3_—MeOH as the eluent to afford pure **7** (1.87 g, 75.7%) as a yellow syrup: *R*_f_ = 0.39 [10:1 (*v*/*v*) CHCl_3_—MeOH]; IR (NEAT): 3294 (ν_O-H_), 2924 (ν_C-H_), 1748 (ν_C=O_, ester), 1660 (ν_C=O_, amide I), 1557 (δ_N-H_, amide II), 1236 (ν_C-O_), 1043 (ν_C-O-C_) cm^−1^; ^1^H-NMR (500 MHz, CDCl_3_): δ 6.80 (d, 1 H, *J*_2,NH_ = 9.30 Hz, NH), 5.11 (dd, 1 H, *J*_2,3_ = 10.0 Hz, *J*_3,4_ = 9.30 Hz, H-3), 5.07 (t, 1 H, *J*_4,5_ = 9.60 Hz, H‒4), 4.77 (d, 1 H, *J*_1,2_ = 8.60 Hz, H‒1), 4.26 (dd, 1 H, *J*_5,6b_ = 4.65 Hz, *J*_6a,6b_ = 12.3 Hz, H‒6b), 4.12 (q, 1 H, H‒2), 4.12 (dd, 1 H, *J*_5,6a_ = 2.60 Hz, H‒6a), 3.90 (dt, 1 H, *J* = 12.65 Hz, *J* = 2.48 Hz, OCH_a_), 3.86–3.60 (m, 12 H, H-5, OCH_2_ × 5, OCH_b_), 3.08 (br s, 1 H, OH), 2.09, 2.02, 2.01, and 1.98 (each s, 12 H, COCH_3_ × 4) (^1^H-MNR spectrum can be found in Supplementary Materials); ^13^C-NMR (100 MHz, CDCl_3_): δ 171.77, 170.94, 170.94, and 169.48 (C=O × 4), 102.05 (C-1), 73.88 (C-3), 72.50 (OCH_2_), 71.71 (C-5), 71.67 (OCH_2_), 70.69 (OCH_2_), 70.25 (OCH_2_), 68.78 (C-4), 68.69 (OCH_2_), 62.30 (C-6), 61.39 (OCH_2_), 54.01 (C-2), 23.12 (NCO*C*H_3_), 20.94, 20.89 and 20.75 (CO*C*H_3_ × 3).

#### 3.2.6. 8-*O*-(2-Acetamido-3,4,6-tri-*O*-acetyl-2-deoxy-β-d-glucopyranosyl)-3,6-dioxaoctanoic Acid (**8**)

An aqueous 15% solution of NaHCO_3_ (19 mL) was added to a solution of alcohol **7** (5.58 g, 11.6 mmol) in acetone (18 mL) with stirring at 0 °C. KBr (0.28 g, 2.3 mmol) and TEMPO (36.4 mg, 0.23 mmol) were added to the mixture. Trichloroisocyanuric acid (5.42 g, 23.2 mmol) was then added slowly over a period of 20 min at 0 °C. The mixture was warmed to room temperature and stirred for 4.5 h, and then propan-2-ol was added. The mixture was filtered on celite and treated with 15 mL of satd aq NaHCO_3_. The aqueous phase was washed with portions of CHCl_3_, treated with 1 M aq HCl and brine, and extracted twice with CHCl_3_. The organic layers were combined and dried over anhyd MgSO_4_, and the solvent was evaporated after filtration on celite to yield the corresponding carboxylic acid **8**, which was used for the next step without further purification: *R*_f_ = 0.40 [5:1 (*v*/*v*) CHCl_3_—MeOH]; IR (NEAT) 3323 (ν_O-H_), 2931 (ν_C-H_), 1745 (ν_C=O_, ester), 1732 (ν_C=O_, carboxy group), 1643 (ν_C=O_, Amide I), 1556 (δ_N-H_, Amide II), 1373 (δ_O-H_), 1234 (ν_O-H_, carboxy group), 1041 (ν_C-O-C_) cm^−1^; ^1^H-NMR (400 MHz, CDCl_3_): δ 6.59 (d, 1 H, *J*_2,NH_ = 9.20 Hz, NH), 5.15 (t, 1 H, *J*_2,3_ = 10.2 Hz, *J*_3,4_ = 9.48 Hz, H-3), 5.07 (t, 1 H, *J*_4,5_ = 9.65 Hz, H‒4), 4.87 (d, 1 H, *J*_1,2_ = 8.52 Hz, H‒1), 4.27 (dd, 1 H, *J*_5,6b_ = 4.60 Hz, *J*_6a,6b_ = 12.2 Hz, H‒6b), 4.14 (dd, 1 H, *J*_5,6a_ = 2.24 Hz, H-6a) 4.06 (q, 1H, H-2), 3.94–3.61 (m, 11 H, H-5 & OCH_2_ × 5), 2.09, 2.02, 2.01, and 1.97 (each s, 12 H, COCH_3_ × 4) (^1^H MNR spectra can be found in Supplementary Materials); ^13^C-NMR (100 MHz, CDCl_3_): δ 172.01, 171.47, 171.47, 171.19, and 169.56 (C=O × 5), 101.87 (C-1), 73.34 (C-3), 71.77 (C-5), 71.38 (OCH_2_), 70.40 (OCH_2_), 68.81 (OCH_2_), 68.55 (C-4), 68.25 (OCH_2_), 62.38 (C-6), 54.20 (C-2), 25.44 (OCH_2_), 23.10 (NCO*C*H_3_), 20.94, 20.90 and 20.78 (CO*C*H_3_ × 3); MALDI-TOF MS calcd for [M + Na]^+^: 515.169. Found: *m/z* 516.101, [M + K]^+^: 532.143. Found: *m/z* 532.083.

#### 3.2.7. 8-*O*-(2-Acetamido-3,4,6-tri-*O*-acetyl-2-deoxy-β-d-glucopyranosyl)-3,6-dioxaoctanoic Acid 4-vinybenzylamide (**9**)

To a stirred solution of carboxylic acid **8** (4.0 g, 8.11 mmol) and AMN-Str **3** (1.24 g, 9.31 mmol) in dichloromethane (20 mL) was added DMT-MM (2.69 g, 9.72 mmol) and the resulting suspension was stirred at room temperature under a N_2_ atmosphere for 3.5 h. The reaction mixture was poured into ice-cold water and extracted with EtOAc. The organic layer was successively washed with satd aq NaHCO_3_ and brine. The organic layer was dried over anhyd MgSO4 and concentrated in vacuo. The residue was purified by silica gel chromatography with 50:1 (*v*/*v*) CHCl_3_—MeOH as the eluent to give styrene derivative **9** (3.82 g, 77.7%) as a colorless syrup: *R*_f_ = 0.48 [10:1 (*v*/*v*) CHCl_3_—MeOH]; IR (NEAT) 3306 (ν_N-H_), 3086, 3007 (ν_C-H_, Ph), 2937 (ν_C-H_), 1748 (ν_C=O_, ester), 1667 (ν_C=O_, Amide I), 1651 (ν _C=C_), 1537 (δ_N-H_, Amide II), 1234 (νC-O), 1043 (ν_C-O-C_) cm^−1^; ^1^H-NMR (400 MHz, CDCl_3_): δ 7.39 (d, 2 H, *J*_vic_ =8.1 Hz, Ph), 7.27 (d, 2 H, *J*_vic_ = 8.3 Hz, Ph), 7.23 (s, 1 H, N*H*CH_2_), 6.71 (dd, 1 H, *J_cis_* = 10.9 Hz, *J_trans_* = 17.6 Hz, C*H*=CH_2_), 6.30 (d, 1 H, *J*_2,NH_ = 8.52 Hz, N*H*Ac), 5.75 (dd, 1 H, *J*_gem_ = 0.60 Hz, *trans* of CH_2_=), 5.35 (dd, 1 H, *J*_2,3_ = 10.5 Hz, *J*_3,4_ = 9.34 Hz, H-3), 5.26 (dd, 1 H, *cis* of CH_2_=), 5.03 (t, 1 H, *J*_4,5_ = 9.72 Hz, H-4), 4.79 (d, 1 H, *J*_1,2_ = 8.36 Hz, H‒1), 4.49 (d, 2 H, *J*_vic_ = 5.92 Hz, NHC*H_2_*), 4.24 (dd, 1 H, *J*_5,6b_ = 4.66 Hz, *J*_6a,6b_ = 12.3 Hz, H‒6b), 4.19 (d, 1 H, *J*_gem_ = 15.7 Hz, NCOCH_b_), 4.13 (d, 1 H, NCOCH_a_), 4.10 (dd, 1 H, *J*_5,6a_ = 2.36 Hz, H-6a), 3.88 (m, 1 H, OCH_b_), 3.71–3.60 (m, 9 H, H-2, H-5, OCH_b_, OCH_2_ × 3), 2.07, 2.01, 2.01, 1.89 (each s, 12 H, COCH_3_ × 4) (^1^H-MNR spectrum can be found in Supplementary Materials); ^13^C-NMR (100 MHz,CDCl_3_): δ 170.86, 170.65, 170.42, and 169.59 (C=O × 4), 136.43 (CH=), 128.17 (Ph), 126.66 (Ph), 114.27 (CH_2_=), 100.60 (C-1), 72.30 (C-3), 71.87, 71.03, 70.89, 70.70 and 70.52 (C-5, OCH_2_ × 4), 68.86 (C-4), 68.62 (OCH_a,b_), 62.26 (C-6), 55.15 (C-2), 42.75 (NCH_2_), 23.29 (NCOCH_3_), 20.89, 20.84, and 20.77 (COCH_3_ × 3); MALDI-TOF MS calcd for [M + H]^+^: 609.265. Found: *m/z* 609.228, [M + Na]^+^: 631.247. Found: *m*/*z* 631.242, [M + K]^+^: 647.221. Found: *m*/*z* 647.225.

#### 3.2.8. 8-*O*-(2-Acetamido-2-deoxy-β-d-glucopyranosyl)-3,6-dioxaoctanoic acid 4-vinybenzylamide (NAG-Str) (**10**) 

Sodium methoxide (53.2 mg, 0.98 mmol) was added to a solution of acetate **9** (2.0 g, 3.29 mmol) in MeOH (20 mL) at room temperature under a N_2_ atmosphere and the reaction mixture was stirred for 1.5 h. When complete disappearance of the acetate **9** on TLC was observed, IR-120B (H^+^) was added to the mixture. The suspension was filtered off and the filtrate was evaporated in vacuo to yield the corresponding NAG-Str **10** (1.48 g, 98.9%) as a colorless syrup: *R*_f_ = 0.41 [65:25:4 (*v*/*v*/*v*) CHCl_3_—MeOH—H_2_O]; IR (NEAT) 3312 (ν_O-H_), 3086, 2999 (ν_C-H_, Ph), 2922 (ν_C-H_), 1651 (ν_C=O_, Amide I), 1556 (δ_N-H_, Amide II), 1298 (ν_C-O_), 1072 (ν_C-O-C_) cm^−1^; ^1^H-NMR (400 MHz, D_2_O): δ 7.49 (d, 2 H, *J*_vic_ = 8.12 Hz, Ph), 7.32 (d, 2 H, *J*_vic_ = 8.08 Hz, Ph), 6.78 (dd, 1 H, *J_cis_* = 11.0 Hz, *J_trans_* = 17.7 Hz, CH=CH_2_), 5.85 (d, 1 H, *trans* of CH_2_=), 5.31 (d, 1 H, *cis* of CH_2_=), 4.50 (d, 1 H, *J*_1,2_ = 8.44 Hz, H-1), 4.45 (s, 2 H, NHC*H_2_*), 4.13 (s, 2 H, COCH_2_), 3.92 (dd, 1 H, *J*_5,6b_ = 4.40 Hz, *J*_6a,6b_ = 12.6 Hz, H-6b), 3.92 (m, 1 H, OCH_b_), 3.67 (m, 9 H, H-2, H-6a, OCH_a_, OCH_2_ × 3), 3.55 (m, 2 H, H-3 and H-4), 2.02 (s, 3 H, COCH_3_) (^1^H-MNR spectrum can be found in Supplementary Materials); ^13^C-NMR (100 MHz, D_2_O): δ 174.42 (C=O, NAc), 172.47 (C=O, N*C*OCH_2_), 137.43 (Ph), 136.68 (Ph), 136.24 (CH=), 127.76 (Ph), 126.48 (Ph), 114.34 (CH_2_=), 101.00 (C-1), 75.86 (C-3), 73.87 (C-5), 70.53 (OCH_2_), 69.91 (C-4), 69.61 (OCH_2_ × 2), 69.54 (OCO*C*H_2_), 68.85 (OCH_2_), 60.75 (C-6), 55.49 (C-2), 42.28 (NCH_2_), 22.16 (NCO*C*H_3_).

### 3.3. Radical Polymerization

A solution of NAG-Str monomer **10** and an appropriate amount of acrylamide (AAm) in deionized water was deaerated under reduced pressure for a few minutes, and then tetramethylethylenediamine (TEMED) (0.2 equivalent molar for **10**) and ammonium persulfate (APS) (0.1 equivalent molar for **10**) were added. The mixture was stirred at room temperature and diluted with 1 M aq pyridine-acetic acid buffer (pH 5.6). The viscous solution was dialyzed against distilled water, followed by lyophilization to provide the corresponding white powdery glycopolymers **11a**–**11d**. The results of the radical polymerization are summarized in Table 1.

### 3.4. Biological Evaluations of Glycopolymers for WGA

Measurement of the fluorescence emission spectra and excitation spectra were carried out in a Teflon-stoppered cuvette (12.5 mm in width × 45 mm height) containing 3.0 mL of a sample. The slit width of the excitation and the emission was 5.0 nm, and the scan speed was medium. The sensitivity was high, and the interval of sampling was 3.0 nm. Emission spectra of WGA induced by excitation at 295 nm were uncorrected and were recorded with a Shimadzu RF-5300PC fluorescence spectrophotometer (Shimadzu Corp., Kyoto, Japan). The cuvette was mounted in a thermostated holder, and measurement was carried out at 4 °C in order to eliminate the effect of nonspecific binding on the spectra. The concentration of WGA was estimated to be 0.65 μM using the absorption coefficient at 280 nm (E_280_^1%^ = 15.0 in 50 mM Tris-HCl buffer, 0.15 M NaCl, pH 7.4) [35].

## 4. Conclusions

In conclusion, chloromethylstyrene was efficiently converted into the desired functional monomers by a combination of azide displacement, reduction and condensation with appropriate functional substrates. The assembly of the functional monomers by means of radical polymerization was performed using a GlcNAc monomer (NAG-Str) as a model monomer, and biological interactions between the polymers and a protein were demonstrated. This methodology can be applied to the preparation of various functional monomers and the corresponding polymers including the reversible deactivation radical polymerization (RDRPs) [41,42,43,44] and microwave-assisted polymer syntheses [45,46].

## Figures and Tables

**Table 1:** Results of the coupling reaction of AMN-Str **3** and biotin.

Reagent	Solvent	Temp (°C)	Time (h)	Yield (%)
DIC	DMF	Rt	on	0
EDC	DMF	Rt	5	0
BOP	CH_3_CN	Rt	3	36 ^1^
BOP	DMF	Rt	1.5	17 ^1^
DMT-MM	DMF	Rt	2	91

^1^ including a trace of inseparable byproducts.

**Table 2:** Results of polymerizations of NAG-Str **10** with or without acryl amide (**AAm**).

Monomer Ratio 10:AAm	Polymer	Time (h)	Solvent	Total Yield ^1^ (%)	Polymer Composition ^2^ x:y:n	Sugar Content (wt%)	$\bar{Mw}\;{\;}^{3}\;(\mathrm{kDa})$	*D* ^4^
1:0	**11a**	69	H_2_O	71	1:0:6.5 × 10 ^2^	100	315	1.13
1:4	**11b**	24	H_2_O	81	1:4:6.2 × 10 ^2^	63	472	1.01
1:10	**11c**	24	H_2_O	99	1:11:1.5 × 10 ^2^	38	183	2.52
1:20	**11d**	24	H_2_O	90	1:22:1.5 × 10 ^2^	24	297	1.34

^1^ Total yields were calculated on the basis of the quantities of monomers used. ^2^ Polymer compositions of sugar unit:acrylamide unit were estimated on the basis of the ^1^H-NMR results. ^3^ The weight-average molecular weights were estimated by size-exclusion chromatography in 0.3 M aq NaCl solution using a Shodex GF-510HQ column. Calibration curves were obtained using pullulan standards (5.9, 11.8, 22.8, 47.3, 112, 212, 404, and 788 kDa, Shodex P-82). ^4^ Dispersity (*D*) were calculated on the basis of ($\bar{Mw}$)/($\bar{Mn}$).

**Table 3:** Results of the binding assays for WGA on the basis of fluorescence measurements.

Compounds	Δλ (nm)	|Δ*F*′/*F*_0_| (%)	*K_a_* (M^−1^)	Relative Potency
**GlcNAc**	0	5	8.2 × 10^3^	1
**10**	-	90	5.0 × 10^4^	6
**11a**	−14.2	33	1.8 × 10^5^	22
**11b**	−14.2	46	3.7 × 10^5^	45
**11c**	−13.0	50	3.5 × 10^5^	43
**11d**	−16.0	58	4.2 × 10^5^	51

## Supplementary Materials

The following are available online, Figure S1: ^1^H-NMR spectrum of DSL-Str **4**, Figure S2: ^1^H-NMR spectrum of BTN-Str **5**, Figure S3: ^1^H-NMR spectrum of compound **7**, Figure S4: ^1^H-NMR spectrum of compound **8**, Figure S5: ^1^H-NMR spectrum of compound **9**, Figure S6: ^1^H-NMR spectrum of NAG-Str **10**.
