# Supplementary material for: Preparation of Functional Monomers as Precursors of Bioprobes from a Common Styrene Derivative and Polymer Synthesis
Source: Molecules. 2018 Nov 4;23(11):2875. doi: 10.3390/molecules23112875 (PMC6278513; doi:10.3390/molecules23112875)

Figure S1. ^{1}H NMR spectrum of DSL-Str **4**

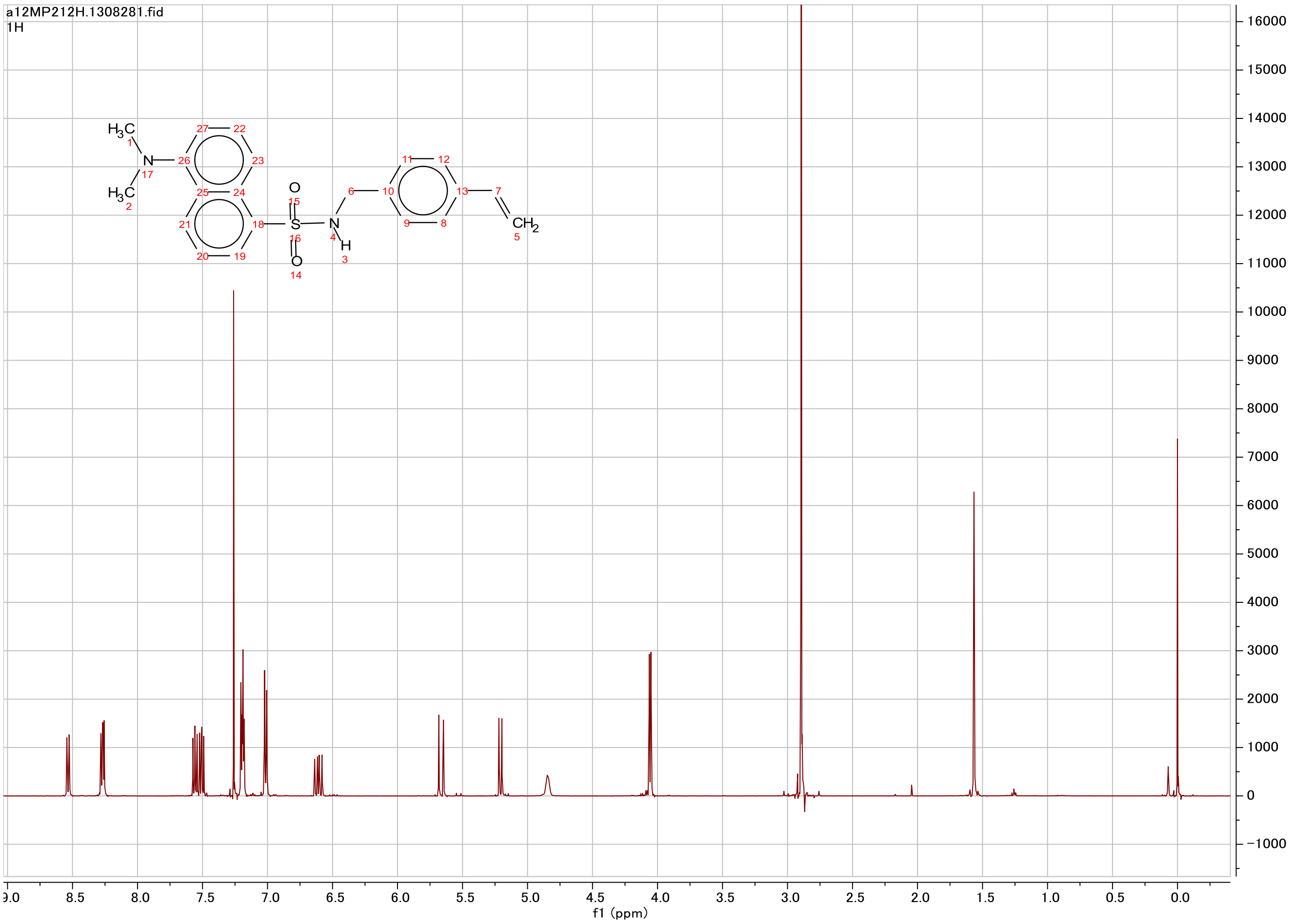

Figure S2. ^{1}H NMR spectrum of BTN-Str **5**

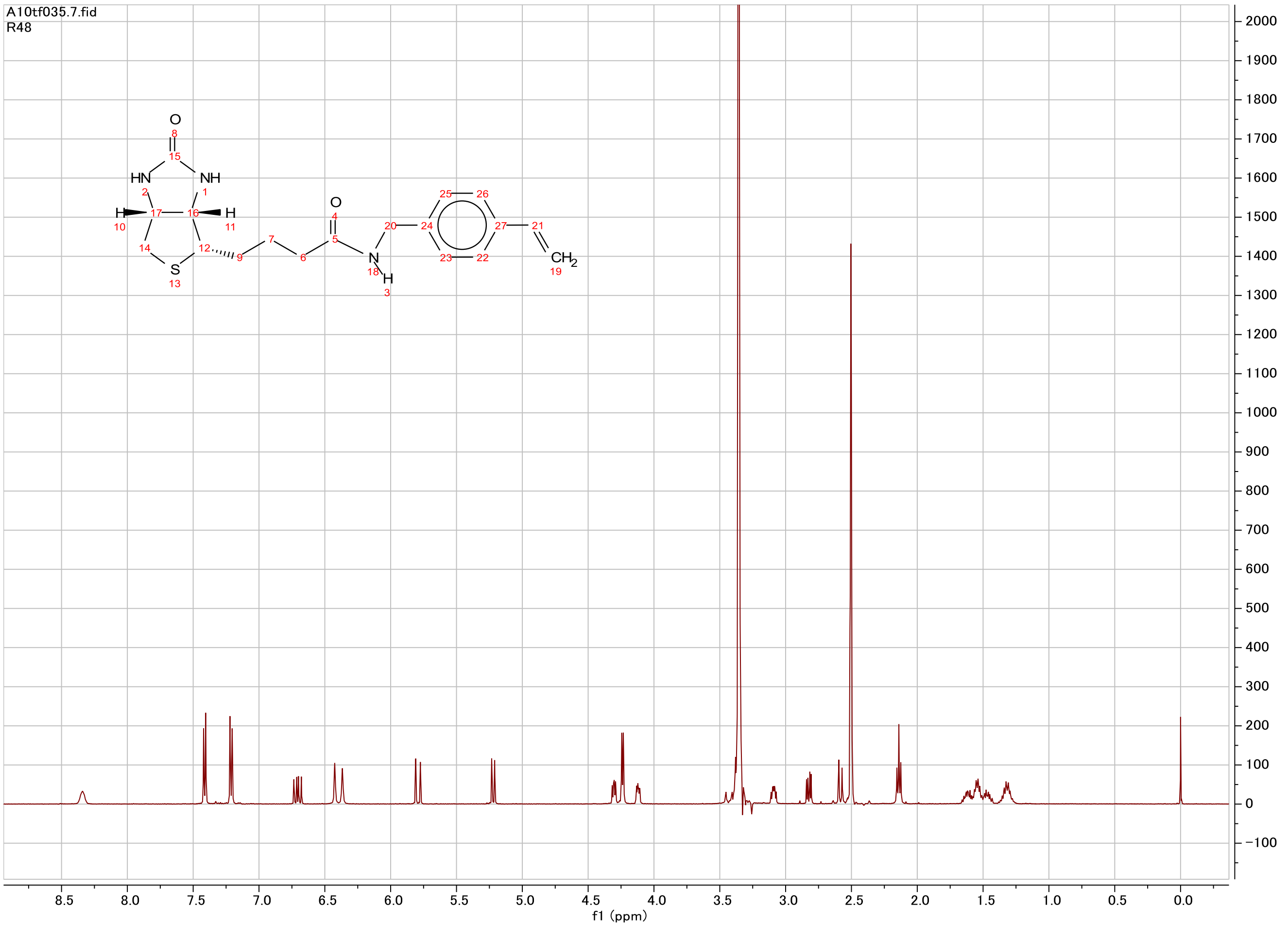

Figure S3. ^{1}H NMR spectrum of Compound **7**

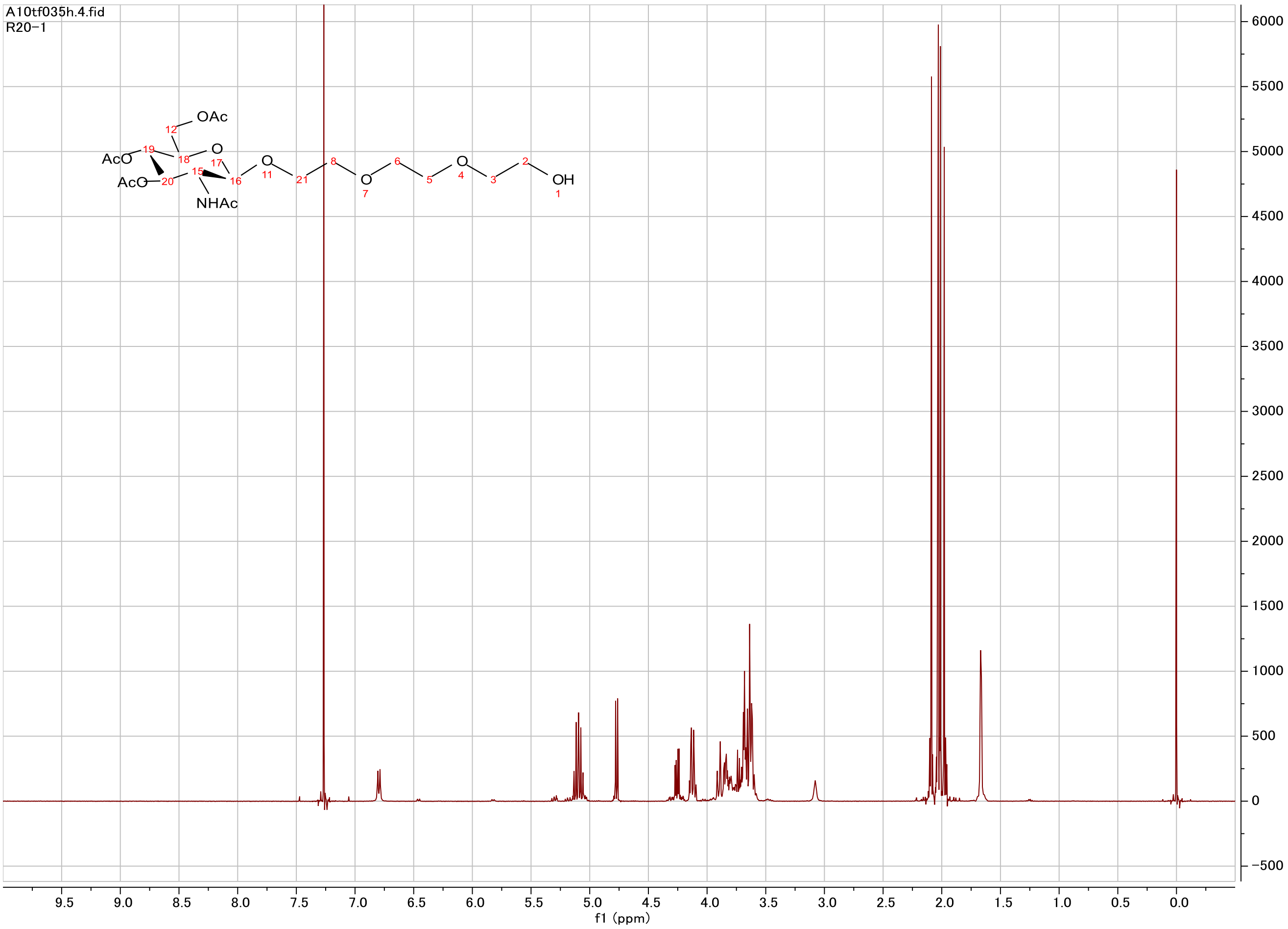

Figure S4. ^{1}H NMR spectrum of Compound **8**

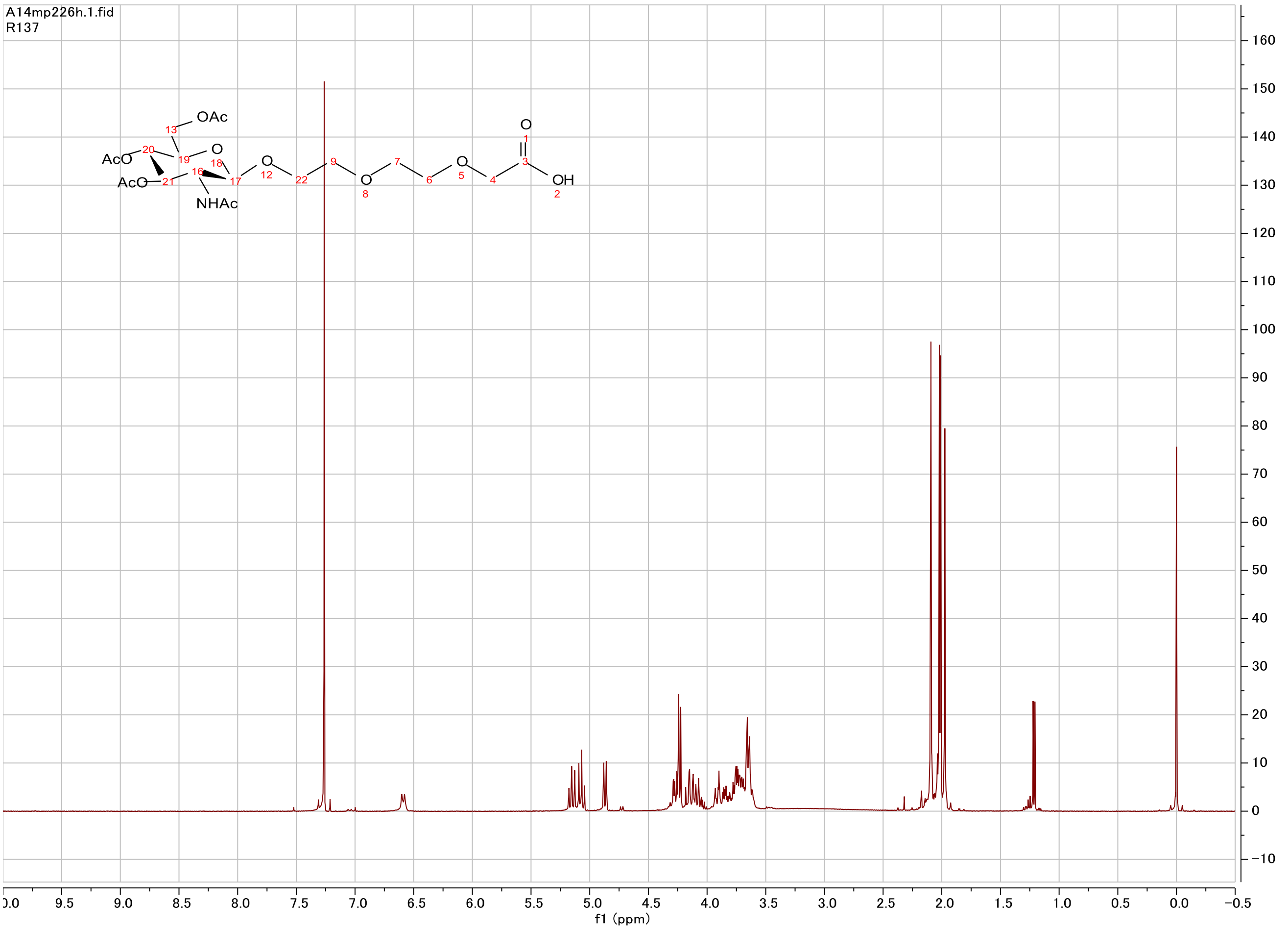

Figure S5. ^{1}H NMR spectrum of Compound **9**

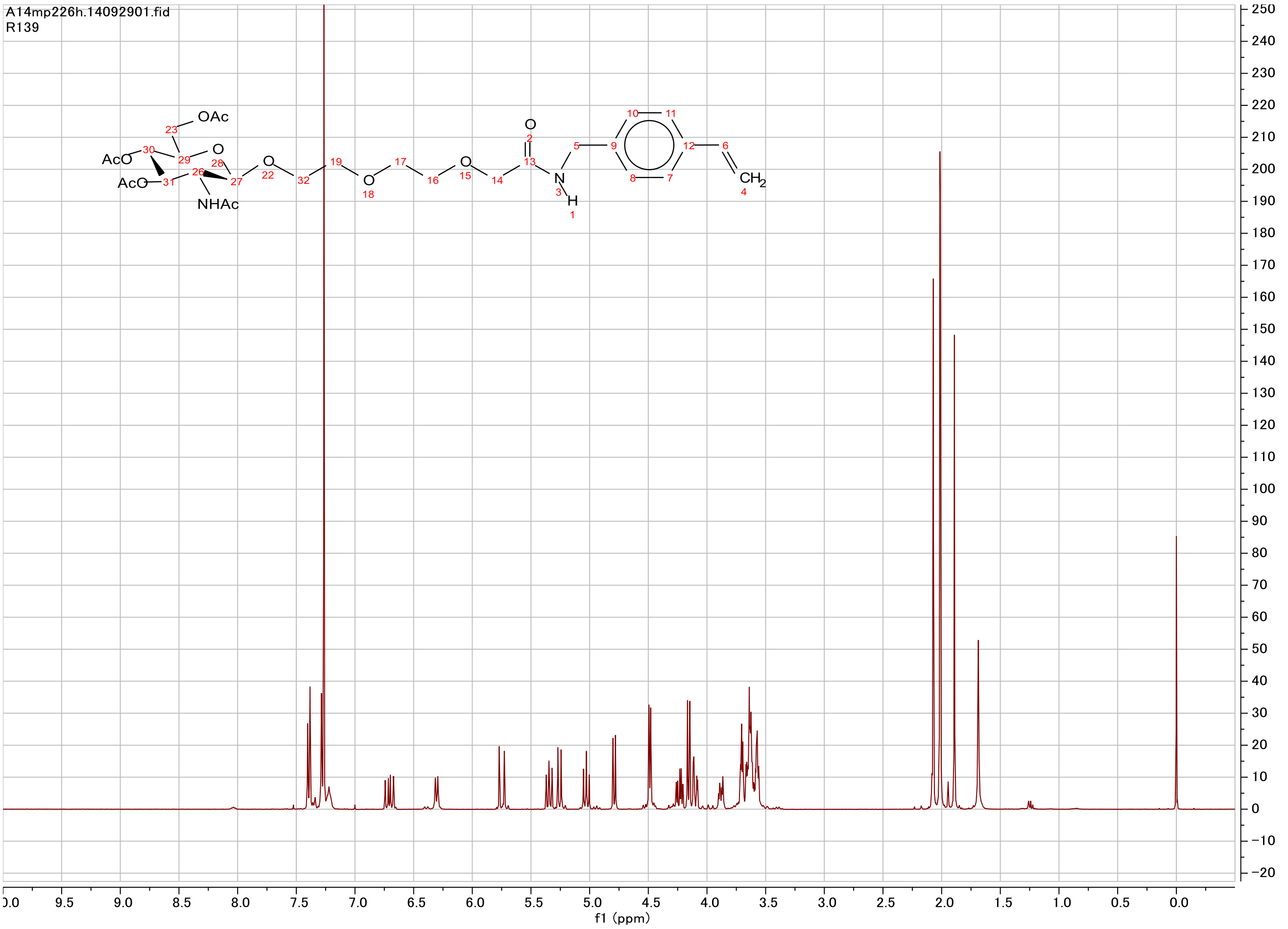

Figure S6. ^{1}H NMR spectrum of NAG-Str **10**

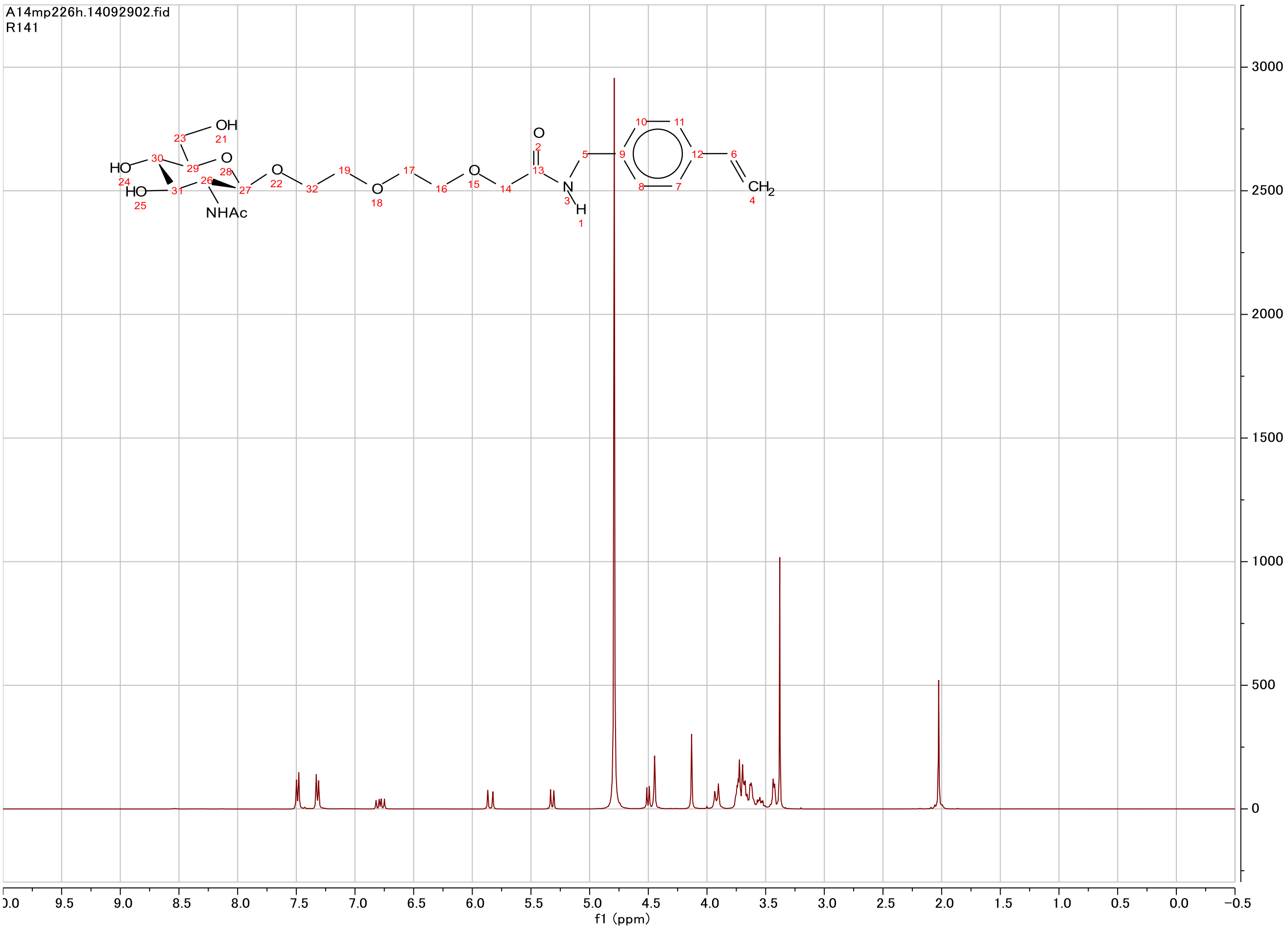

Supplement: Supplementary file 1 [file molecules-23-02875-s001.pdf]
